# Understanding AI’s Role in Endometriosis Patient Education and Evaluating Its Information and Accuracy: Systematic Review

**DOI:** 10.2196/64593

**Published:** 2024-10-30

**Authors:** Juliana Almeida Oliveira, Karine Eskandar, Emre Kar, Flávia Ribeiro de Oliveira, Agnaldo Lopes da Silva Filho

**Affiliations:** 1 Department of Women's Health Federal University of Minas Gerais Belo Horizonte Brazil; 2 Department of Medicine Pontifical Catholic University of Paraná Curitiba Brazil; 3 Department of Obstetrics & Gynecology Cam and Sakura City Hospital Istanbul Turkey

**Keywords:** endometriosis, gynecology, machine learning, artificial intelligence, large language models, natural language processing, patient-generated health data, health knowledge, information seeking, patient education

## Abstract

**Background:**

Endometriosis is a chronic gynecological condition that affects a significant portion of women of reproductive age, leading to debilitating symptoms such as chronic pelvic pain and infertility. Despite advancements in diagnosis and management, patient education remains a critical challenge. With the rapid growth of digital platforms, artificial intelligence (AI) has emerged as a potential tool to enhance patient education and access to information.

**Objective:**

This systematic review aims to explore the role of AI in facilitating education and improving information accessibility for individuals with endometriosis.

**Methods:**

This review followed the Preferred Reporting Items for Systematic reviews and Meta-Analyses (PRISMA) guidelines to ensure rigorous and transparent reporting. We conducted a comprehensive search of PubMed; Embase; the Regional Online Information System for Scientific Journals of Latin America, the Caribbean, Spain and Portugal (LATINDEX); Latin American and Caribbean Literature in Health Sciences (LILACS); Institute of Electrical and Electronics Engineers (IEEE) Xplore, and the Cochrane Central Register of Controlled Trials using the terms “endometriosis” and “artificial intelligence.” Studies were selected based on their focus on AI applications in patient education or information dissemination regarding endometriosis. We included studies that evaluated AI-driven tools for assessing patient knowledge and addressed frequently asked questions related to endometriosis. Data extraction and quality assessment were conducted independently by 2 authors, with discrepancies resolved through consensus.

**Results:**

Out of 400 initial search results, 11 studies met the inclusion criteria and were fully reviewed. We ultimately included 3 studies, 1 of which was an abstract. The studies examined the use of AI models, such as ChatGPT (OpenAI), machine learning, and natural language processing, in providing educational resources and answering common questions about endometriosis. The findings indicated that AI tools, particularly large language models, offer accurate responses to frequently asked questions with varying degrees of sufficiency across different categories. AI’s integration with social media platforms also highlights its potential to identify patients’ needs and enhance information dissemination.

**Conclusions:**

AI holds promise in advancing patient education and information access for endometriosis, providing accurate and comprehensive answers to common queries, and facilitating a better understanding of the condition. However, challenges remain in ensuring ethical use, equitable access, and maintaining accuracy across diverse patient populations. Future research should focus on developing standardized approaches for evaluating AI’s impact on patient education and exploring its integration into clinical practice to enhance support for individuals with endometriosis.

## Introduction

Endometriosis, a chronic gynecological condition characterized by the presence of endometrial-like tissue outside the uterus, affects 6% to 10% of reproductive-aged women [1,2]. This disease has a high degree of morbidity due to chronic pelvic pain and infertility [1,2]. It is likely polygenic and multifactorial, but the exact pathogenic mechanisms remain unclear [3,4]. Endometriosis not only causes debilitating symptoms such as chronic pelvic pain, dysmenorrhea, and infertility but also poses substantial challenges in diagnosis, management, and patient education [3,4].

Quality of life in women with endometriosis is a widely debated topic within the medical community, as it is influenced by the unpredictability of symptom progression, varying treatment outcomes, and the psychosocial impact of living with a chronic illness [5-9]. Recent studies have highlighted the association of endometriosis with psychiatric comorbidities, such as anxiety, eating disorders, and mood disorders [10]. This exacerbates the multifaceted burden faced by women with endometriosis, highlighting the need to measure and understand their uncertainties and questions related to the condition.

In the digital age, where information dissemination and patient empowerment are increasingly facilitated through online platforms, social media has emerged as a prominent avenue for individuals seeking support, information, and community engagement [11]. The exponential growth of social media use, coupled with advancements in artificial intelligence (AI), presents new opportunities and challenges in how patients access and interpret information related to their health. Access to information through social platforms by patients needs to be assessed, since guidance based on high quality evidence is necessary [11].

AI technologies such as natural language processing and machine learning algorithms have revolutionized data analysis capabilities, enabling the extraction of meaningful insights from vast amounts of unstructured data generated on social media platforms [5-9]. These tools not only enhance the efficiency of processing large datasets but also offer potential solutions to mitigate the risks of misinformation and improve the dissemination of evidence-based medical knowledge [12].

Despite these advancements, significant gaps remain in understanding how AI can best serve the needs of patients with endometriosis, particularly in facilitating informed decision-making, enhancing health literacy, and addressing the unique informational needs of diverse patient populations. The ethical implications of AI-driven interventions in patient education and support must be carefully considered to ensure equitable access and privacy protection [12,13].

Therefore, this systematic review aims to critically evaluate the current literature on the role of AI in patient education and information access for endometriosis. By synthesizing existing evidence, we seek to elucidate the potential benefits, challenges, and future directions of AI integration in improving the quality of care and support for individuals affected by this complex condition.

## Methods

### End Points, Eligibility, and Selection Criteria

This systematic review was performed according to the recommendations of the Cochrane Collaboration [14] and the Preferred Reporting Items for Systematic reviews and Meta-Analyses (PRISMA) statement [15].

Studies were only included in this review if they addressed (1) knowledge about endometriosis evaluated through AI or (2) AI platform answers on common questions regarding endometriosis. We excluded studies (1) with patients who had not received an endometriosis diagnosis, (2) in which the evaluation was not performed using AI, and (3) that did not apply language models for acquiring answers or knowledge about the disease. Time of follow-up, language of publication, and type of study were not limited as a means of approaching as many subjects as possible. We collected and analyzed common data from the studies for comparison purposes.

### Search Strategy and Data Extraction

We systematically searched PubMed, Embase, the Regional Online Information System for Scientific Journals of Latin America, the Caribbean, Spain and Portugal (LATINDEX), Latin American and Caribbean Literature in Health Sciences (LILACS), Institute of Electrical and Electronics Engineers (IEEE) Xplore, and Cochrane Central Register of Controlled Trials in May 2024. The search was updated in September 2024. We used the following Medical Subject Heading (MeSH) terms: “endometriosis” and “artificial intelligence.” We acknowledge that search syntaxes vary across databases, and as such, each search strategy was carefully tailored to the specific requirements of each database to ensure comprehensiveness. Notably, we were able to either increase the number of included papers or maintain those identified by the previous strategy by using the search strategies detailed in Multimedia Appendix 1, Table S1. Screening was carried out independently by 2 authors (JAO and KE) following the predefined search criteria. Furthermore, both authors performed data collection for the included studies independently. Any conflicts were resolved by consensus among the authors.

### Quality Assessment

To assess the quality of the included studies, we used tools from the Joanna Briggs Institute (JBI) [16]. Studies that used AI to analyze social media responses [11] were evaluated using the Critical Appraisal Checklist for Analytical Cross-Sectional Studies, while studies that investigated AI-generated responses [12,13] were evaluated using the Critical Appraisal Checklist for Textual Evidence [17]. Although these tools are widely accepted, we acknowledge that they were not specifically designed for AI.

## Results

### Study Selection and Description of Included Studies

**Figure 1:**
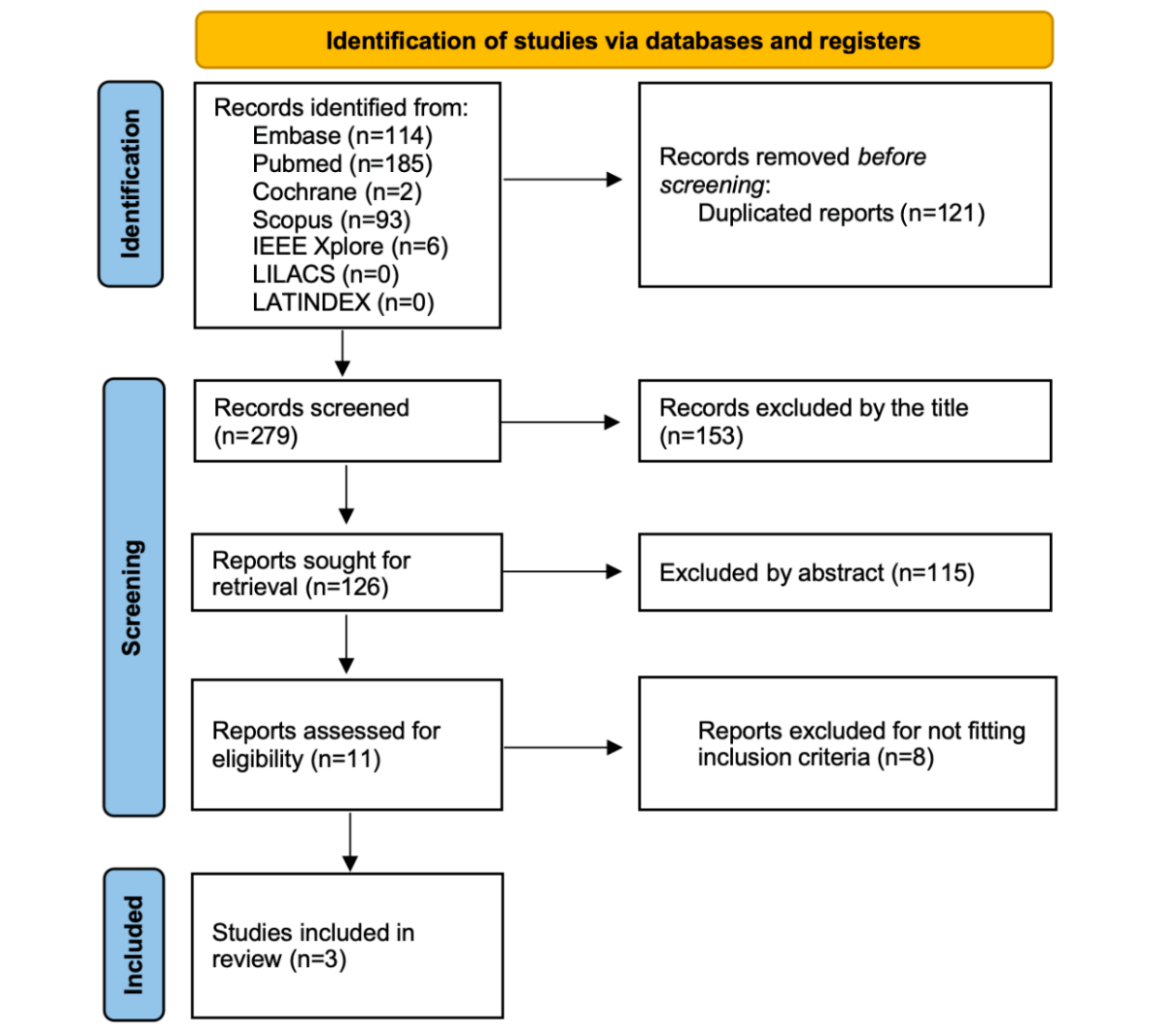
Screening flow diagram. IEEE: Institute of Electrical and Electronics Engineers; LATINDEX: Regional Online Information System for Scientific Journals of Latin America, the Caribbean, Spain and Portugal; LILACS: Latin American and Caribbean Literature in Health Sciences.

The initial search yielded 400 results. Duplicate records and ineligible studies were removed, and 11 studies remained that were fully reviewed based on the inclusion criteria. Of these, 3 studies were included, including 1 abstract (Figure 1); 2 evaluated language models and consequently had no patients [12,13], while 1 evaluated patients’ knowledge in the last 11 years through comments, evaluating over 31,144 online users [11]. Of the 3 studies, 2 had similar designs involving expert analysis of chatbot responses [12,13], and 1 was a sentiment analysis and topic modeling study with observational data (user-generated content) [11].

The use of AI was not described in much detail. One of the studies cited use of the “WordNetLemmatize” function and “PorterStemmer” functions from the *Natural Language Toolkit* package and the *SpellChecker* package in Python, for linguistic correction and reducing word forms [9]. For topic definitions, the same study applied the LDAMulticore algorithm, a probabilistic generative model. The algorithm identified the main topics of the comments and posts and the 10 words most related to each topic [9]. Unfortunately, no further research data were available in the manuscript or supplemental material, or by request.

Additional study characteristics are reported in Table 1.

**Table 1:** Main characteristics of the studies.

Author, year	AI^a^ model used	Aim	Main area	Patients, n	Main findings
Cohen et al [12], 2024 (abstract)	ChatGPT (GPT-4; (OpenAI), Claude (Anthropic), and Bard (Google)	To assess and compare the chatbots’ information accuracy for its answers about endometriosis	Patients’ knowledge through AI	—^b^	Experts’ validations of the answers were graded; the answers were observed to be mostly accurate yet insufficient for commonly raised inquiries.
Goel et al [11], 2023	Machine learning association with BERT^c^; sentiment analysis assisted by Python	To identify, with the Reddit application programming interface, discussion topics and themes to guide health care professionals and researchers on women’s needs	Patients’ needs	31,144	Social media might help to diminish the overlap between research priorities and topics discussed in social media regarding endometriosis. Surgery, advice, diagnosis, mental health, and pain should be further discussed.
Ozgor and Simavi [13], 2024	ChatGPT	To assess the quality of ChatGPT FAQ^d^ answers about endometriosis	Patients’ knowledge through AI	—	Of all FAQs, 91.4% (n=71) were properly answered. Accuracy was highest in the symptom and diagnosis category (91.1%), and lowest in the treatment category (81.3%).

^a^AI: artifcial intelligence.

^b^Not applicable.

^c^BERT: Bidirectional Encoder Representations from Transformers.

^d^FAQ: frequently asked question.

### AI and Social Media Platforms

To better understand the needs of women of all ages with endometriosis, a study was conducted on Reddit, a platform where people join communities focused on a common topic, over the previous 136 months (11.3 years). A total of 45,693 posts and 357,498 comments were analyzed; 92.09% of the posts were associated with negative sentiments in a sentiment analysis. Most posts were related to surgery (16.85%), followed by questions or advice (16.12%); diagnoses (12.34%); and feelings, depression, or pain (6.4%). As for the areas with the most comments, they involved sex and intimacy. Importantly, an exploratory approach used manual analysis of 3000 randomly selected posts, finding that 0.5% were made by individuals concerned about loved ones with endometriosis, reflecting the impact of endometriosis on society. Furthermore, after 2011 there was an increase in the number of posts and comments, which might be related to higher awareness of endometriosis [11]. A limitation was that Reddit users are mainly from the United States, Australia, and India, whereas health care assistance differs among countries and the questions and doubts of these users might not represent women with endometriosis worldwide [18].

### AI and Large Language Models

Frequently asked questions (FAQs), or common questions, on endometriosis were used by 2 studies for assessment of the accuracy of language models [12,13]. One study [13] using ChatGPT applied a wide range of questions created based on questions identified in social media and online platforms (n=41), as well as scientific questions (n=40) based on the European Society of Human Reproduction and Embryology (ESHRE) endometriosis guidelines. The 81 questions compiled were classified as being about general information (n=20), symptoms and diagnosis (n=17), treatment (n=16), prevention (n=15), and complications (n=13). An experienced endometriosis gynecologist gave scores of 1 to 4 for each answer provided by ChatGPT: 1 for completely true answers, 2 for accurate answers with insufficient data, 3 for answers containing correct and incorrect information, and 4 for completely incorrect answers [13].

A total of 91.4% (n=71) of the FAQs were considered accurate and sufficient, while no answers were considered completely incorrect. ESHRE-based questions were mostly considered to have completely true answers (67.5%). ChatGPT had the highest accuracy in the symptom and diagnosis category (94.1%) and the lowest in the treatment category (81.3%). Each question was asked twice, and if the answers were divergent, it was considered as having negative reproducibility. The reproducibility rate was 100% for questions related to prevention, symptoms and diagnosis, and complications. The lowest rate was for treatment questions (81.3%) and ESHRE-based questions (70%) [13].

The other study approached 3 large language models (LLMs) and applied 10 FAQs on endometriosis. The answers were compared to guidelines and expert opinions and were rated and averaged between 3 gynecologists. They were graded similarly to the previous study. The answers were graded as 1 if completely incorrect, 2 if mostly incorrect but somewhat correct, 3 if mostly correct but somewhat incorrect, 4 if correct but inadequate, and 5 if correct and comprehensive. Among 3 LLMs, Bard had better average scores than ChatGPT or Claude. Most answers were considered correct but inadequate, and only 1 ChatGPT and Bard answer was graded as 5 by all experts [12]. Only this study shared the questions applied to chatbots.

### Quality Assessment

The quality assessment revealed considerable variation among the studies. The study that used AI to analyze social media responses [11] received a moderate quality rating (62%) based on the JBI Critical Appraisal Checklist for Analytical Cross-Sectional Studies. This rating was primarily due to the lack of clear identification of patients with and without endometriosis and the inability to explore confounding factors. In contrast, the studies that evaluated AI-generated responses [12,13] were assessed using the JBI Critical Appraisal Checklist for Textual Evidence and were classified as low quality (<50%), mainly due to insufficient information about the sources of opinions and limited presentation of the data. While the tools used were not specifically designed for evaluating studies involving AI, they were the most appropriate options available.

## Discussion

### Principal Findings

LLMs are frequently used as a source of knowledge on health conditions due to their ability to provide rapid and concise responses [19]. It has been found that they provide answers to questions about cardiovascular disorders [20] and pediatric urology that are accurate and consistent with the subspecialty guidelines, [21] and that they can interpret radiological imaging with a low error rate [22]. Yet, there are still doubts concerning the use of AI in medical practice and as a source of patient education [23].

Social media is often used to identify patients’ struggles and for patient education through experience exchange [8,9]. As observed, many patients use social media for education, but previous studies have observed that inaccurate information is 30-fold higher than accurate information [24]. Unfortunately, it has been shown that research- and education-related posts attract less engagement than posts related to emotional support [7,8].

When compared to online chat, chatbots have become a faster and safer way of acquiring information on health conditions [25]. Because of this, their answers have been compared to human scores and test results. A study applied United States Medical Licensing Examination (USMLE) step 1, 2, and 3 questions to ChatGPT and achieved over 60% accuracy without prior training [26,27]. ChatGPT has also been used for ophthalmology resident examination questions and was found to obtain scores similar to those of ophthalmology residents [28].

Chatbots can be a particularly useful asset for patients with endometriosis, since diagnosis is often delayed by 6 to 14 years from first symptoms [29,30]. This delay is commonly due to patients and physicians normalizing the symptoms, which stems from a limited understanding of endometriosis etiology and restricted access to specialized care [29-31]. Additionally, diagnosing endometriosis can be challenging and requires, regardless of the method, evaluation by specialized physicians [32,33]. The diagnosis can be performed either through the standard diagnostic method (laparoscopy combined with abdominal cavity exploration and histological biopsy [34]) or through secondary methods, such as magnetic resonance imaging and transvaginal ultrasound [33]. All these factors play a role in treatment and diagnosis delay, with clinical implications such as chronic pain, reduction in quality of life, and higher treatment cost [30]. The exact impact on fertility is still unknown [32-34], but it has been noted that women with a short delay were less likely to have infertility [29]. These factors enhance the need for further evaluation of online support groups’ access to information [34] and AI responses, especially in places with low access to specialized care.

AI is already being used in medical practice, and responsible AI is vital to maximize the relationship between health care professionals and patients [35,36], not only for better understanding of their feelings [11], but also for understanding whether most information provided to them is correct [12,13]. A study reported that only 25% of general practitioners feel adequately informed about endometriosis, though those with gynecology qualifications (58.9%) or continuing medical education in the field (19.6%) had better awareness [37]. In the general population, women’s knowledge about endometriosis in generally low. A study in a high-income country had only 4.5% reporting very good knowledge and about one-third indicating sufficient or good knowledge about endometriosis [38]. In a lower-middle-income country, an endometriosis knowledge score was measured in women from the general population, and it was found that they had a mean score of 4.2 of 10 [39]. Patients’ use of AI for education and clarification is common and might enhance knowledge about endometriosis, especially whenever medical explanations are insufficient or not easily available [11]. AI is a part of digital health and using it for patients’ benefit is needed [34,40,41].

This review emphasizes the need for applying AI to data analysis and increasing the amount of evaluated data. Furthermore, analysis of ChatGPT responses is important since many health professionals use it as a supplementary source of information so that patients can obtain a better understanding of the disease. Additionally, it increases the time health professionals can devote to their patients by creating spare time.

This review has limitations. First, not all questions used in the studies were made available. Secondly, some countries might use chatbots more often, as is known to be the case for Reddit [18], and only 1 study cited tested reproducibility [13]. Furthermore, the questions and posts or comments could not be separated by source: people with endometriosis, friends or family of people with endometriosis, health professionals, or people who are simply curious.

Although we rigorously followed the Cochrane and PRISMA guidelines throughout the process, the protocol for this systematic review was not registered, which may affect the transparency and replicability of the findings. Registration on appropriate platforms was considered but could not be completed due to technical difficulties, approval delays, and the temporary suspension of new submissions. Additionally, the exploratory nature of the review, particularly in the field of AI, and the specificities of the study design, which did not fully meet the criteria of these platforms, also contributed to this limitation.

The absence of specific tools for evaluating studies involving AI was an important limitation of this study. Although we used the JBI tools, we acknowledge that they do not fully capture the particularities of AI [42]. The development of PROBAST (Prediction model study Risk Of Bias Assessment Tool)-AI, a tool currently being developed using the Delphi method, will be essential for improving quality assessment in future AI studies, offering greater precision and relevance [42]. However, this tool was not available at the time this article was completed [42].

The limited scope of this review restricts a comprehensive understanding of the topic. Future studies should adopt standardized methodologies, with validated questions and greater access to data from online users with endometriosis, in order to expand the understanding of the impact of AI on patient education for this condition.

### Conclusions

Patient education can be better assessed through AI evaluation and might provide insights on endometriosis for patients with the disease, as well as for professionals in other specialties. The use of AI for endometriosis should be under a gynecologist’s supervision and might be beneficial for diagnosis and follow-up insights. LLMs cannot guide clinical decisions, and these should be based on current endometriosis guidelines.

## Appendix

Multimedia Appendix 1 Table S1 and S2.

Multimedia Appendix 2 Preferred Reporting Items for Systematic reviews and Meta-Analyses (PRISMA) checklist.

## Abbreviations

AI: artificial intelligence
ESHRE: European Society of Human Reproduction and Embryology
FAQ: frequently asked question
IEEE: Institute of Electrical and Electronics Engineers
JBI: Joanna Briggs Institute
LATINDEX: Regional Online Information System for Scientific Journals of Latin America, the Caribbean, Spain and Portugal
LILACS: Latin American and Caribbean Literature in Health Sciences
LLM: large language model
MeSH: Medical Subject Heading
PRISMA: Preferred Reporting Items for Systematic reviews and Meta-Analyses
PROBAST: Prediction model study Risk Of Bias Assessment Tool
USMLE: United States Medical Licensing Examination
