# Supplementary material for: Understanding AI’s Role in Endometriosis Patient Education and Evaluating Its Information and Accuracy: Systematic Review
Source: JMIR AI. 2024 Oct 30;3:e64593. doi: 10.2196/64593 (PMC11561426; doi:10.2196/64593)
Supplement: Multimedia Appendix 1 [file ai_v3i1e64593_app1.docx]

**Supplemental Material**

**Oliveira JA, et al**

**Table S1**. Search terms

| **Platform** | **Search terms** |
| --- | --- |
| Pubmed, Embase, Cochrane, LILACS, LATINDEX, IEEE Xplore, and Scopus | (Endometriosis OR endometrioma OR endometriomas) AND ("artificial intelligence" OR "Intelligence, Artificial" OR "Computational Intelligence" OR "Intelligence, Computational" OR "Machine Intelligence" OR "Intelligence, Machine" OR "Computer Reasoning" OR "Reasoning, Computer" OR "AI (Artificial Intelligence)" OR "Computer Vision Systems" OR "Computer Vision System" OR "System, Computer Vision" OR "Systems, Computer Vision" OR "Vision System, Computer" OR "Vision Systems, Computer" OR "Knowledge Acquisition (Computer)" OR "Acquisition, Knowledge (Computer)" OR "Knowledge Representation (Computer)" OR "Knowledge Representations (Computer)" OR "Representation, Knowledge (Computer)") |

**Table S2.** Studies that appeared to meet inclusion criteria, but that were excluded

| **Author/year** | **Title** | **Excluded by reading the...** | **Reason for exclusion** |
| --- | --- | --- | --- |
| Balogh et al, 2024(1) | FEMaLe: The use of machine learning for early diagnosis of endometriosis based on patient self-reported data-Study protocol of a multicenter trial | Abstract | Protocol: absence of results |
| Goldstein and Cohen, 2023(2) | Self-report symptom-based endometriosis prediction using machine learning | Full text | Patients were not diagnosed with endometriosis |

References:

1. Balogh DB, Hudelist G, Bļizņuks D, Raghothama J, Becker CM, Horace R, Krentel H, Horne AW, Bourdel N, Marki G, Tomassetti C, Kirk UB, Acs N, Bokor A. FEMaLe: The use of machine learning for early diagnosis of endometriosis based on patient self-reported data-Study protocol of a multicenter trial. PLoS One. 2024 May 9;19(5):e0300186. doi: 10.1371/journal.pone.0300186. PMID: 38722932; PMCID: PMC11081275.
2. Goldstein A, Cohen S. Self-report symptom-based endometriosis prediction using machine learning. Sci Rep. 2023 Apr 4;13(1):5499. doi: 10.1038/s41598-023-32761-8. Erratum in: Sci Rep. 2024 May 7;14(1):10443. doi: 10.1038/s41598-024-61280-3. PMID: 37016132; PMCID: PMC10073113.
